# Last Visit to Hospitals

**Published:** 1920-05-08

**Authors:** 


### Last Visit to Hospitals.

Voluntary hospitals all over the world are incalculably the poorer to-day by the death of Sir Henry Burdett, which occurred on Thursday last week. Six months ago he paid his last visit to hospitals and came to Scotland; although he was then suffering from over-work and over-strain, he visited the principal hospitals in Edinburgh and Glasgow, and did much to establish the systematic method of securing funds for the maintenance of the voluntary hospitals, and hoped to see them thoroughly sound financially within a year. Burdett's work for hospitals was so extensive that it cannot be epitomised. He has done more for hospitals than any man of his generation, and it is to be hoped that eventually full justice will be done to it. He was a great personality, and a man of high ideals. He will be sadly missed by all who have the interests of hospitals at heart, especially by nurses, as he took the deepest interest in every movement for their welfare and was at all times their true friend.—D. J. M.

